# Tooth shade selection using digital and visual method under light correcting device

**DOI:** 10.6026/973206300212437

**Published:** 2025-08-31

**Authors:** Angela Justin, Pushkar Gupta, Sneha S. Mantri, Aditi M. Paranjpe, Indrapal Singh Kanwar, Ravi Prakash Tiwari

**Affiliations:** 1 Department of Prosthodontics and Crown & Bridge, Hitkarini Dental College and Hospital, Jabalpur, Madhya Pradesh, India

**Keywords:** Esthetic dentistry, shade selection, spectrophotometer, visual shade matching

## Abstract

A segmental shade discrepancy between digital and visual methods in the maxillary central incisors is of interest. Sixty-four participants aged 18-45 with intact central incisors were assessed. Shade determination at the cervical, middle and incisal thirds was performed using the VITA Easy shade V spectrophotometer and the 3D Master shade guide under a standardized light-correcting device. Distinct shade variations were noted between the digital and visual methods, reflecting the inherent polychromatic nature of natural dentition. Thus, we show the importance of adopting a combined, segmental approach to shade selection to enhance accuracy and ensure superior esthetic results in clinical practice.

## Background:

The selection of shades for anterior teeth is among the most crucial elements of aesthetic dentistry, since it is a major factor in shaping the overall appearance and harmony of dental restorations [1]. Natural-looking restorations require accurate shade selection, which depends on understanding illumination, translucency and the visual behavior of dental materials [2]. Human brain recognises almost one million shades while precise technology can recognise up to 10 million. Electronic instruments can recognise almost 100,000 different dental tints, while the human eye can only recognise 1% of these hues [3]. Teeth are polychromatic in nature, which means they can show varied tints throughout distinct segments. This is especially noticeable in the anterior teeth, where the cervical, middle and incisal thirds can exhibit significant colour differences [4]. Hue, value and chroma are the three basic parameters that make up tooth colour, which is an intricate mix [5]. Tooth colour is assessed using subjective and objective methods. Visual shade matching is the most common clinical technique due to its simplicity, but it lacks consistency and repeatability [6]. Instrumental methods offer more reliable results by reducing subjectivity, while hybrid approaches combine both for greater accuracy. Several factors affect shade selection, with lighting being crucial [7]. Natural daylight, with its balanced spectrum, is ideal for shade matching. Light directly influences colour perception thus using consistent, calibrated lighting sources in clinics such as daylight-simulating lamps or handheld tools helps minimize variability [8]. Light-correcting devices provide a steady light source, allowing doctors to make more precise shade assessments. The optimal lighting situation for selecting tooth hues is one with a Colour Rendering Index (CRI) of at least 90 and a colour temperature between 5500 K and 6500 K [9]. Consequently, a precise light source and spectrum dispersion are necessary for accurate and repeatable colour matching. Although visual shade matching using shade guides is widely accepted, instrumental methods have increasingly gained recognition and appreciation. In the realm of dental colour matching, spectrophotometers are among the most precise, rapid and versatile tools available [10]. It uses optical technology to assess the colour of teeth by analysing the light reflected from their surface, breaking it down into its spectral components and analysing them to determine the precise colour. When selecting a shade either by visual or instrumental, the teeth must be divided into three sections: the gingival area (which accurately measures dentinal chroma), the middle, and the incisal area (where enamel is denser and ranges from translucent to transparent) [11]. Therefore, it is of interest to evaluate the shade of the cervical, middle and incisal thirds of the maxillary central incisor using a spectrophotometer and the VITA 3D-Master shade guide under a standardized light correction device.

## Material and Methods:

The study was conducted in accordance with the Declaration of Helsinki (2013). All subjects participated voluntarily and provided their written informed consent to participate in this study. The study was approved by the institutional ethical committee of Hitkarini Dental College & Hospital, Jabalpur. An observational comparitive study was carried out in which 128 samples were observed. Participants were informed about the purpose of the study and informed consent was obtained. Tooth shade was assessed objectively using the VITA Easyshade® V spectrophotometer and subjectively using the VITA 3D-Master shade guide (VITA Zahnfabrik, Germany), under a light-correcting device. Based on specific criteria, participants were selected for the study. The inclusion criteria consisted of healthy individuals with sound maxillary central incisors, dentition and gingiva, no recent bleaching or severe discoloration and no direct or indirect restorations. The exclusion criteria included the presence of restorations, orthodontic fixed appliances involving the central incisors, endodontically treated or non-vital teeth, fractured teeth, veneers and fluorosis. Applying these criteria, 64 participants visiting Hitkarini Dental College and Hospital were selected. Each tooth was assessed in the cervical, middle and incisal thirds, resulting in a total of 192 observations per method. To eliminate discrepancies in the traditional method of shade selection, the clinician had an Ishihara colour vision test in an eye hospital and a certificate was obtained from an expert opthomalogist. This clinical study proposes the null hypothesis that the shade of the cervical, middle, and incisal thirds of the maxillary central incisor does not significantly differ when evaluated using either a digital spectrophotometer or the 3D Master shade guide with a light-correcting device.

## Measurement process:

## Visual method:

A standardized protocol was followed for visual shade selection [9]. A single operator conducted all procedures with participants seated on the same dental chair, facing northern sunlight, using a light-correcting device, set to approximately 5500 Kelvin to ensure proper illumination for accurate colour measurement. Shade matching was done at a distance of 61-183 cm using the VITA 3D-Master Shade Guide. The maxillary central incisor was divided into cervical, middle and incisal thirds [12]. Shade selection began with determining the value (lightness), followed by chroma and hue, comparing the tooth to shade tabs under controlled lighting. To reduce eye fatigue, the operator viewed a grey card between assessments [13]. The shade was selected from the VITA 3D-Master display and was recorded in the master chart for comparison.

## Spectrophotometric method:

The digital shade analysis of the maxillary central incisor was performed using the VITA Easyshade-V spectrophotometer. Participants leaned back in the chair, and the probe tip was positioned at a 90-degree angle [14] on the cervical third of the labial surface of the maxillary central incisor. A minimum of 2mm distance from the tissue margin was maintained. After pressing the measurement button, the probe emitted two rapid beeps to indicate completion. The reflected light was analyzed, and the color shades and ΔE values were displayed. The same procedure was repeated for the middle and incisal thirds. The spectrophotometer was recalibrated after each use (Figure 1 - see PDF).

## Statistical analysis:

Data was entered in Microsoft Excel 2021 for Windows. Frequencies and percentages of variables were calculated. The data of maxillary central incisors teeth shade in cervical, middle and incisal third segments were on ordinal scale. To evaluate agreement for shade between cervical, middle and incisal third segments of maxillary central incisors in 3D master tooth shade guide under light correcting device and spectrophotometric method groups (Intra-group agreement), Fleiss' Kappa was applied. To evaluate agreement for shade between 3D master tooth shade guide under light correcting device and spectrophotometric method groups in cervical, middle and incisal third segments of maxillary central incisors (Inter-group agreement), Weighted Kappa was applied. P value <0.05 was considered statistically significant. Data analyses were performed using MedCalc - version 23.1.7 (MedCalc Software Ltd, Ostend, Belgium) and DATAtab-2025 (DATAtabe.U. Graz, Austria) software.

## Results:

An analysis was conducted to evaluate the agreement in shade selection among the cervical, middle and incisal thirds of maxillary central incisors using the 3D Master Tooth Shade Guide under a light correcting device and spectrophotometer. Table 1 (see PDF) Shows shade agreement across tooth thirds. Table 2 (see PDF) compares percentage shade match of tooth thirds using 3D Master guide under light-corrected and spectrophotometric methods. Table 3 (see PDF) assesses shade agreement between 3D Master (light-corrected) and spectrophotometer across tooth thirds.

## Discussion:

In the present study, the VITA 3D-Master shade guide was employed for visual shade selection due to its enhanced precision and reduced subjectivity compared to traditional systems. Paravina *et al.* (2009) reported that the 3D-Master system provided better matching with natural dentition, a wider color range, and more uniform shade distribution than the Vitapan Classical guide [15]. Consistent shade matching is influenced by individual color perception and lighting conditions. Color vision plays a critical role in esthetic dentistry, and individuals with color vision deficiencies may show reduced shade-matching accuracy. Gokce *et al.* (2010) demonstrated that both color vision deficiency and light source significantly affect shade selection. In this study, the investigator's normal color vision was confirmed via the Ishihara test by an optometrist and ophthalmologist [16]. The GDP Trulite handheld light-correcting device used in this study features a color temperature range close to 5,500 Kelvin and a color rendering index (CRI) greater than 90, aligning with standardized lighting conditions for accurate shade evaluation. Several studies have shown that light-correcting devices significantly improve shade-matching accuracy compared to clinical lighting [17. Prolonged color exposure can fatigue the eye's photoreceptors, causing afterimages and reduced sensitivity. To prevent this, the investigator limited shade selection to three consecutive subjects, with 5-7 second breaks using an 18% neutral gray card to recalibrate color perception [13]. Highlighting the complex interplay of shades within a single tooth, the maxillary central incisor was segmented into cervical, middle, and incisal thirds to capture their full chromatic diversity. Each segment was evaluated individually under a light-correcting device, with shade selection proceeding in the order of value, chroma and hue. This approach is consistent with a previous study [12], which found that the distinct color differences observed across these regions were attributable to variations in enamel thickness, dentin composition, and light transmission. In modern dentistry, objective methods are increasingly prioritized over subjective approaches. The shift from visual technique which is subjective has been largely driven by the inconsistencies in shade selection, which stems from observer bias, varying lighting conditions, and individual differences in color perception. This shift leads to the introduction of advanced electronic devices such as colorimeter, spectrophotometer etc [18]. The VITA Easyshade V was the instrument of choice for shade assessment in this study, to ensure objective shade analysis. According to Kim *et al.* it was the only device to achieve both accuracy and reliability scores around 90% across repeated trials. A spectrophotometer, precisely measures light reflected from or transmitted through teeth, analyzing individual wavelengths to determine value, chroma and hue and converts this data into clinically useful shade information [19]. The spectrophotometer projects halogen light from the tip's periphery onto the tooth for accurate shade analysis. A consistent 90-degree angle between the device and tooth surface is essential, as tilting can cause light distortion and inaccurate readings. In this study, careful positioning at a right angle was maintained to ensure reliable and reproducible results [14, 20].

Table 1 (see PDF) shows segmental shade evaluation of maxillary central incisors, with Fleiss' Kappa values of 0.19 (slight agreement) for visual method and 0.26 (fair agreement) spectrophotometric method. These findings highlight shade variation across the cervical, middle, and incisal thirds. These results align with the study by O'Brien*et al.* which demonstrated significant variations in CIE Lab* values among the three segments of permanent maxillary central incisors. The segmental color variation of maxillary incisors is influenced by tooth structure and light interaction. This was demonstrated in a study by Enabulele *et al.* which explored the polychromatic nature of teeth and concluded that the cervical third appears darker due to thinner enamel and greater dentin influence. The middle third has a balanced mix of enamel translucency and dentin. The incisal third, with the thickest enamel and minimal dentin influence is more translucent and may appear lighter or bluish due to light scattering [5]. The cervical area is also more prone to staining from saliva, plaque, and pigments near the gingival margin, unlike the smoother, less stain-prone middle and incisal thirds-highlighting a natural color gradient across the tooth. This aligns with O'Brien *et al.* who found that the gingival region had the highest L and b values, while the incisal region had the lowest [12]. These findings emphasize the importance of considering regional shade variations to enhance accuracy in aesthetic dental restorations and achieve more natural-looking results. The reliability of conventional and spectrophotometric methods for tooth shade selection has been widely researched, highlighting the importance of accurate shade matching for aesthetically favourable dental restorations. This study also evaluated both methods while adhering to standardized protocols to minimize errors and ensure precise comparisons. Tables 2 and 3 (see PDF) illustrates the agreement between the 3D Master Tooth Shade Guide under a light-correcting device and the spectrophotometric method which was 26.56% (n = 17) in the cervical third (κ = 0.41), 32.81% (n = 21) in the middle third (κ = 0.47), and 34.38% (n = 22) in the incisal third (κ = 0.49), all indicating moderate agreement (P<0.001). The overall agreement was 31.25% (n = 60) with a κ value of 0.46 (moderate agreement) and p value, (P<0.001) indicating a highly significant result. These findings are consistent with Chen *et al.*'s systematic review, which concluded that instrumental methods provide greater accuracy in shade matching [21]. According to Abu-Hossin *et al.* intraoral scanners can help clinical practice workflow. They work well as a supplement to help determine colour [22]. Igiel *et al.* supported the use of color-matching devices to enhance esthetic outcomes [10]. Therefore, relying solely on the visual method can compromise the accuracy of shade selection and overall esthetic outcomes. This study underscores the benefits of incorporating instrumental methods alongside visual techniques for shade selection, emphasizing their combined reliability and precision for a more comprehensive and accurate approach. It also highlights the importance of considering color variations across different segments of the tooth, as these differences are crucial for achieving accurate shade matching. A key limitation of this study is that it does not account for texture, translucency, or surface glosses, which are essential factors in dental aesthetics. Additionally, the reliance on a spectrophotometer, despite its ability to provide objective measurements, poses a challenge due to its high cost and limited accessibility in clinical practice. Furthermore, patient-specific factors such as tooth hydration, enamel thickness, surface texture and the influence of surrounding soft tissues were not considered, despite their impact on shade perception.

## Conclusion:

Both digital and visual methods revealed significant shade variations across the cervical, middle and incisal thirds, underscoring the tooth's inherent polychromatic nature. The overall shade agreement between visual and digital techniques was 31.25%, leading to the rejection of the null hypothesis. Thus, we state the necessity for a comprehensive approach to shade selection in clinical dentistry to enhance precision and esthetic outcomes.

## References

[1] Chu SJ (2011). Shade Matching and Communication in Esthetic Dentistry Second Edition.

[2] Paravina RD, Powers JM (2004). sthetic Color Training in Dentistry.

[3] Jouhar R (2022). Appl Sci..

[4] Hasegawa A (2000). J Prosthet Dent..

[5] Enabulele J (2020). Ann Dent Oral Health..

[6] Brook AH (2007). Int Dent J..

[7] sahin N, Ural C (2024). J Adv Prosthodont..

[8] Clary JA (2016). J Prosthet Dent..

[9] Borse S, Chaware SH. (2020). J Indian Prosthodont Soc..

[10] Igiel C (2017). J Esthet Restor Dent..

[11] Fondriest J (2003). Int. J. Periodontics RestorDent..

[12] O'Brien WJ (1997). Dental Materials..

[13] Snyder T (2010). Shade guides and color visualization. Dent Econ..

[14] Islam M (2024). The Open Dent J..

[15] Paravina RD (2009). J Dent..

[16] Gokce HS (2010). The J Prosthet Dent..

[17] Jouhar R (2022). Int J Environ Res Public Health..

[18] Paul SJ (2004). Inter J Perio Rest Dent..

[19] Kim HK (2018). J Adv Prosthodont..

[20] https://cdn.vivarep.com/contrib/vivarep/media/pdf/4_4673_EasyshadeVOperationManual_20170830220525471.pdf.

[21] Chen H (2012). Quintessence Int..

[22] Abu-Hossin S (2023). Clin Exp Dent Res..

